# Supplementary material for: Vaccine Confidence During Public Health Challenges and Prior to HPV Vaccine Introduction in Mali
Source: Vaccines (Basel). 2025 May 17;13(5):535. doi: 10.3390/vaccines13050535 (PMC12115454; doi:10.3390/vaccines13050535)

**Supplemental Materials for**
**"Vaccine Confidence during Public Health Challenges and Prior to HPV Vaccine Introduction in Mali"**

## Supplemental Figures

**Supplemental Figure S1a**: Map of Bamako CSCOMs & CSREF.

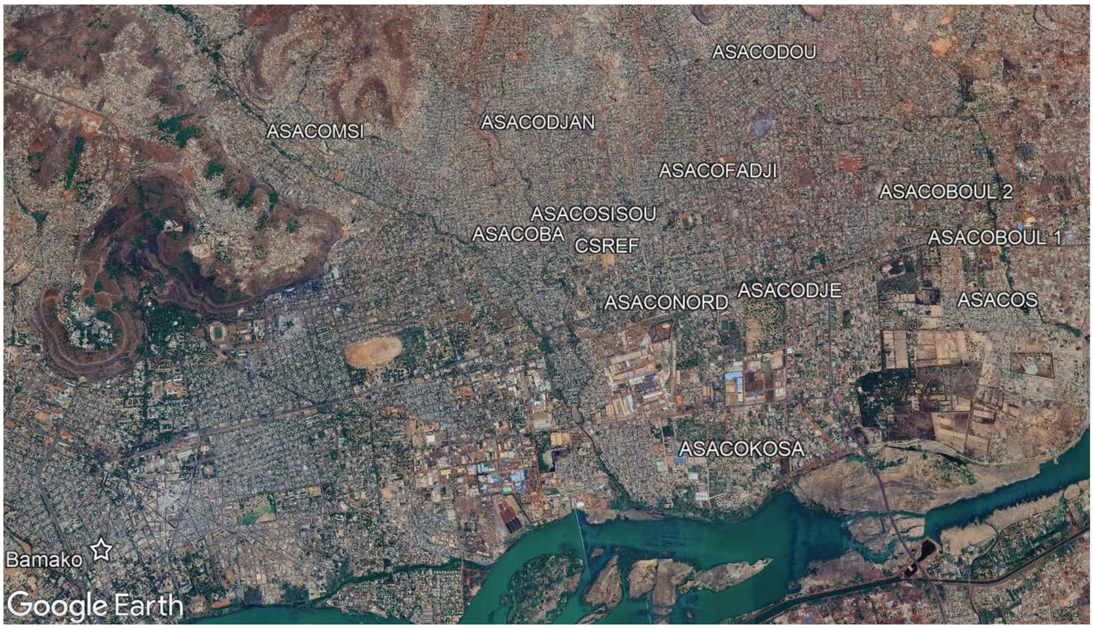


**Supplemental Figure S1b**: Map of Rural CSCOM-ASAKENIERO.

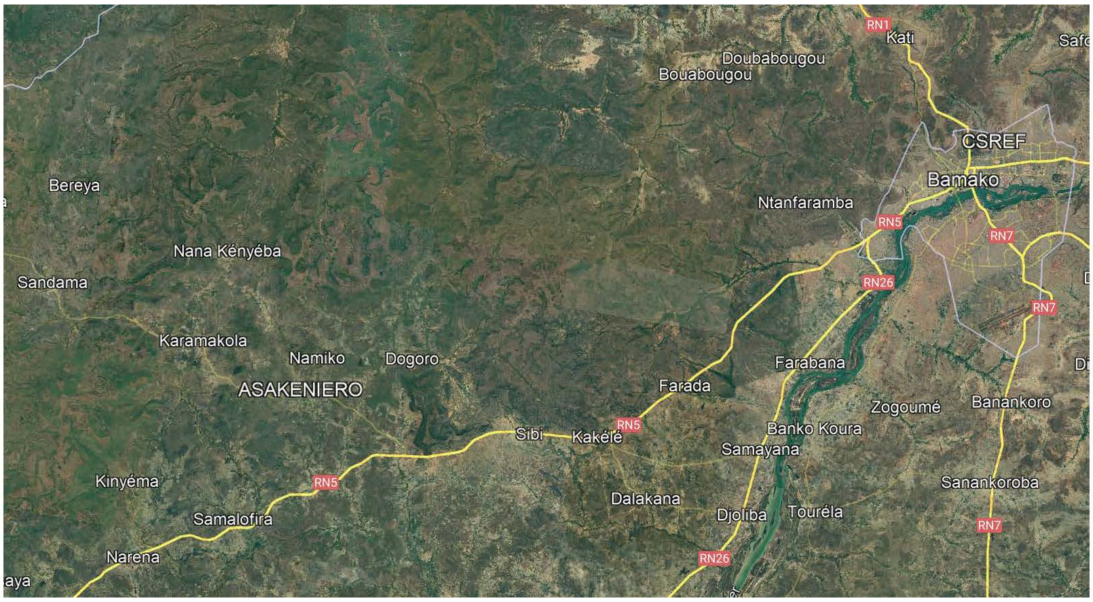

**Supplemental Figure S2**: Posters hung at clinics in French and Bambara.

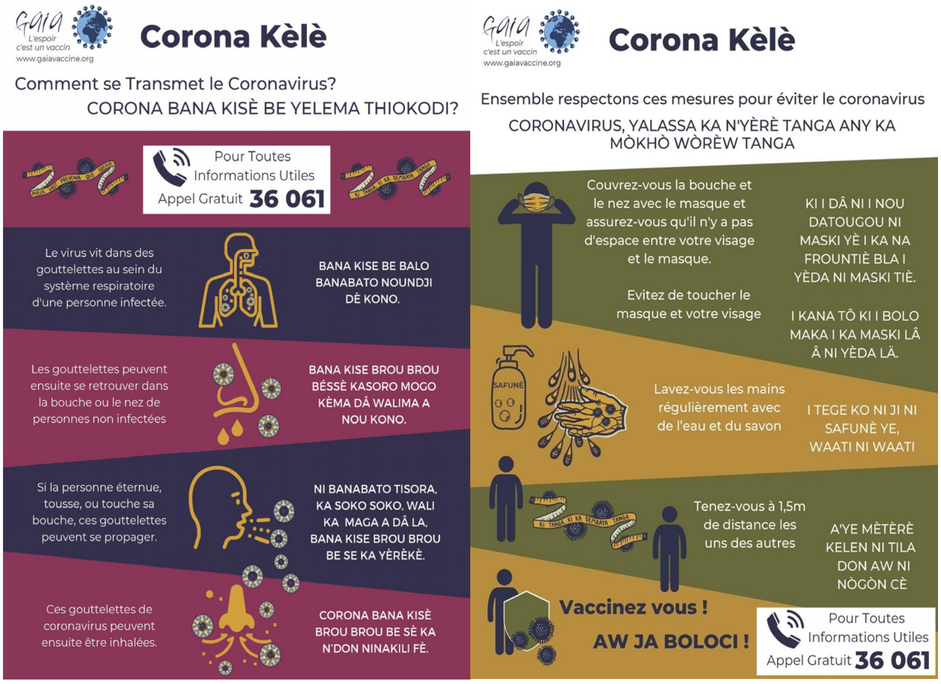


**Radio Advertisement Dialogue**:
The ad begins by asking if you believe in the existence of the coronavirus, and then asked if you have thought about being vaccinated and vaccinating your loved ones. Next the radio hosts said that "GAIA in collaboration with the Ministry of Health is organizing an educational community campaign … a survey is at the heart of the campaign to understand the decision to be vaccinated among 12 CSCOMS in Commune 1 of Bamako and Keniero." Finally it ended with "Better to prevent than to cure" in both Bambara and French

**Story-telling Cloth Design:**
The story-telling cloth was designed by members of the study team (ADG, TC and GDG). It drew inspiration from previous 'story telling cloths' developed by GAIA VF and West African commemorative prints that are often used to promote events such as International Women's Day and World AIDS day. The pattern illustrates the masks (barrier protection), hand washing, and a measuring tape (for "distance"). A virus-like design, that can also be interpreted to show person to person transmission of the virus, is also shown on the cloth. The pattern contains a slogan in French, "Mieux prevenir que guerir" and a local proverb in Bambara, "Banakoubé kafisa ni bana foura kèyé', meaning, "It is better to prevent than to cure"

Two versions were developed in different but complementary colors. GAIA Vaccine Foundation was assisted by Ousmane Sow, a fashion designer based in Dakar Senegal, who provided input on the design (regarding size of the figures, and basic color selections) as well as Genevieve De

Groot, who developed the repeating pattern of designs for the production of the story telling cloth, and for the Corona Kele campaign. The cloth was printed at Batex, in Bamako.

**Survey Design:** The GAIA VF study team created a draft of the community survey using previous GAIA VF studies and the World Bank's 'Mali-COVID-19 High Frequency Phone Survey of Households, 2020' as guides (World Bank. Mali- COVID-19 High Frequency Phone Survey of Households, 2020. Dataset downloaded from www.microdata.worldbank.org). The draft was disseminated to Malian health authorities for comments and review before being finalized and submitted for ethical review in both the United States (E&I/Salus; Study number 21116) and Mali (USTTB; 2021/182/CE/USTTB).

Malian public health partners reviewed and revised drafts of the healthcare provider survey, and the final version was submitted for ethical review in both the United States (E&I/Salus; Study number 21116) and Mali (USTTB; 2021/182/CE/USTTB). The purpose of the survey was explained to all participating HCPs at the beginning of the training. Consent forms were distributed along with copies of the survey to any HCP willing to participate. Participating HCP completed surveys both before and after the training. The pre/post design was used to ascertain retention of the information provided in addition to querying HCP about their general knowledge, attitudes, practices (KAP) related to COVID-19 ,

**Supplemental Figure S3**: HCW wearing the 'story telling cloth'.

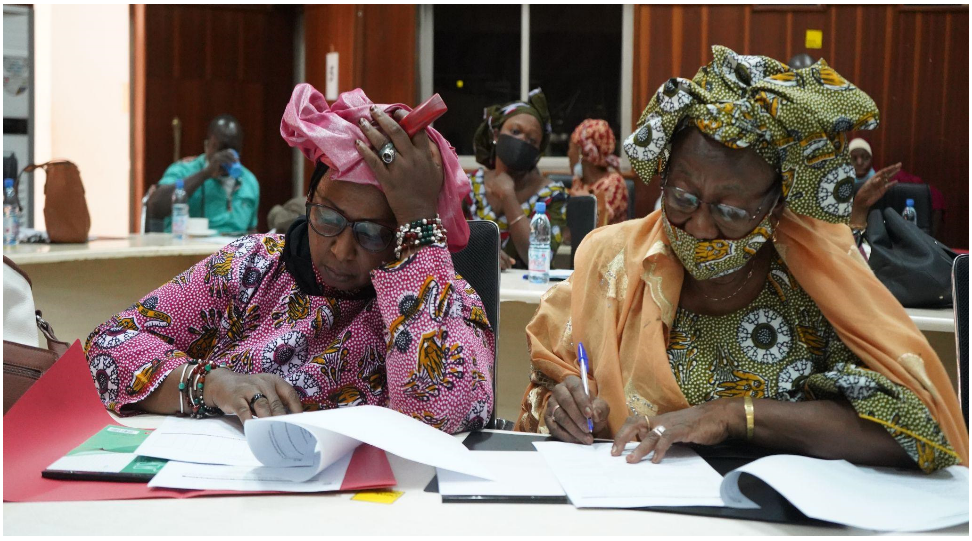

**Supplemental Figure S4:** Healthcare worker Training.

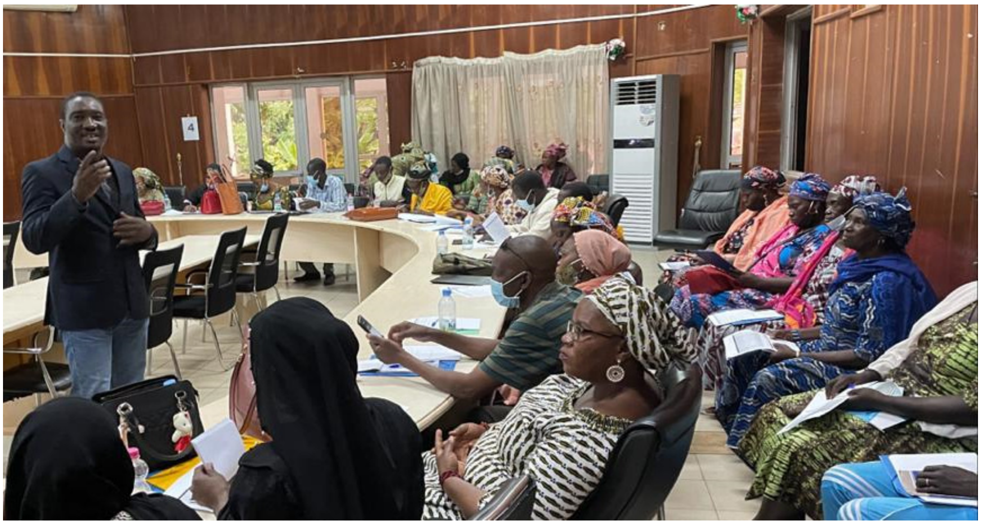

**Supplemental Figure S5**: Exposure to Campaign & Vaccine Confidence.

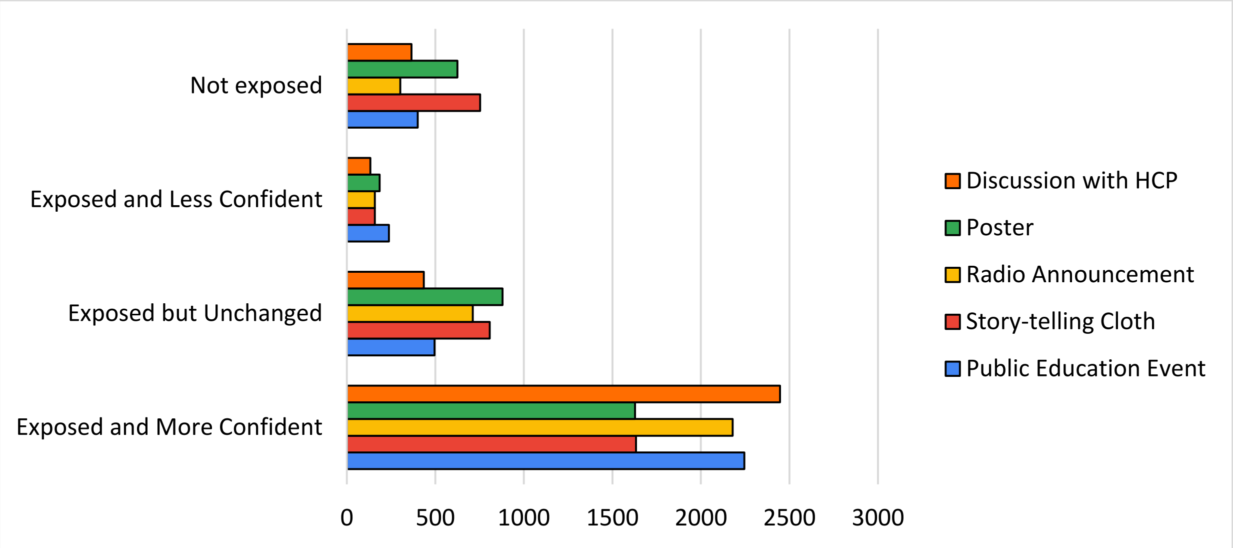


To evaluate whether the outreach activities had a positive or negative survey effect on overall confidence in vaccines, the survey included questions about participation in the intervention and the impact on vaccine confidence. Relatively few people were not exposed to any intervention and even fewer found their confidence reduced after being exposed.

**Supplemental Table S1:** Descriptive Statistics.

| Table S1a: Community Participants | Mean | St. Dev |
|---|---|---|
| Age | 31 | 14.5 |
| | **Number** | **Percent** |
| Female | 2528 | 73% |
| Have Children | 2635 | 76% |
| No Schooling | 863 | 25% |
| Primary Schooling | 768 | 22% |
| Secondary Schooling | 692 | 20% |
| Tertiary Schooling | 605 | 18% |
| University | 482 | 14% |
| **Total Surveyed** | **3445** | |

| Table S1b: Healthcare Providers | Mean | St Dev |
|---|---|---|
| Average Age | 37 | 8.6 |
| | **Number** | **Percent** |
| Female | 112 | 80% |
| Doctor | 18 | 13% |
| Nurse | 48 | 34% |
| Midwife | 57 | 41% |
| Other | 8 | 6% |
| Occupation Not Disclosed | 9 | 6% |
| **Total Surveyed** | **140** | |

**Supplemental Table S2:** Vaccination Advice Sources.

*How often do you trust the following individuals/groups for vaccination advice?*

| | Community Health Providers | Traditional Healers | Government Health Authorities | Local Representatives | Religious Leaders |
|---|---|---|---|---|---|
| Always | 2165 | 320 | 976 | 437 | 508 |
| Often | 759 | 1041 | 1011 | 1130 | 1065 |
| Sometimes | 363 | 888 | 768 | 914 | 857 |
| Never | 127 | 1123 | 633 | 879 | 929 |

| | Actors, Athletes, Etc | Head of the Family | My Father | My Mother | My Friends |
|---|---|---|---|---|---|
| Always | 427 | 1087 | 1202 | 1275 | 786 |
| Often | 933 | 1067 | 942 | 906 | 1077 |
| Sometimes | 825 | 712 | 661 | 655 | 925 |
| Never | 1151 | 484 | 548 | 514 | 532 |

| **Supplemental Table S3:** HCP COVID Survey Answers. | **Number** | **Percent** |
|---|---|---|
| Received a COVID Vaccine | 111 | 79% |
| Had side effects from COVID vaccine | 40 | 29% |
| Believes COVID is a major public health threat | 134 | 95% |
| Believes COVID still exists in Mali | 136 | 97% |
| Believes that COVID survives in hot climates | 116 | 83% |
| Believes official COVID figures match reality | 85 | 61% |
| Believes COVID can be serious | 135 | 96% |
| Knows someone who got seriously ill or died from COVID | 95 | 68% |

Supplemental Figure S6: Vaccine Confidence in Healthcare providers Before & After Training.

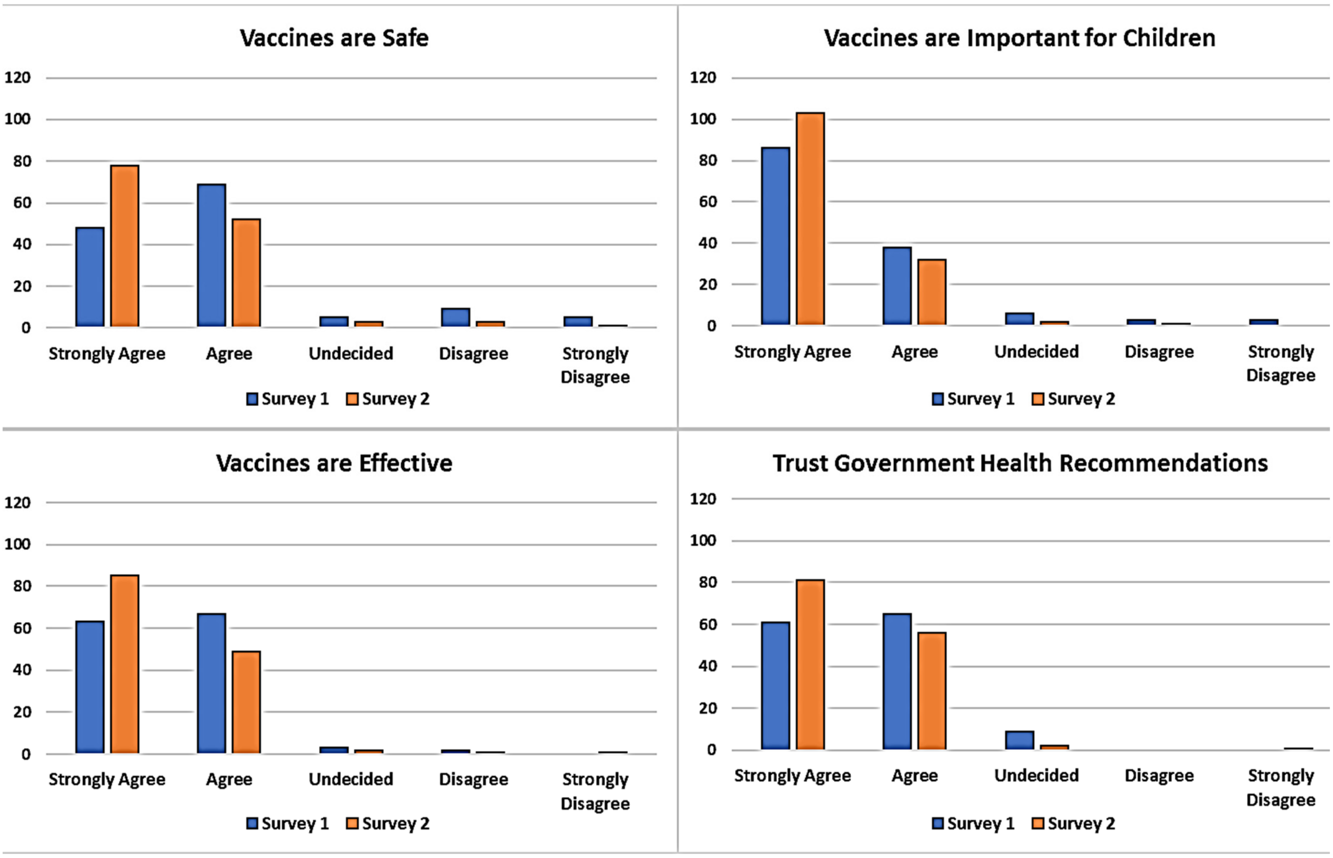

Supplemental Figure S7: HPV and cervical cancer data over 4 projects.

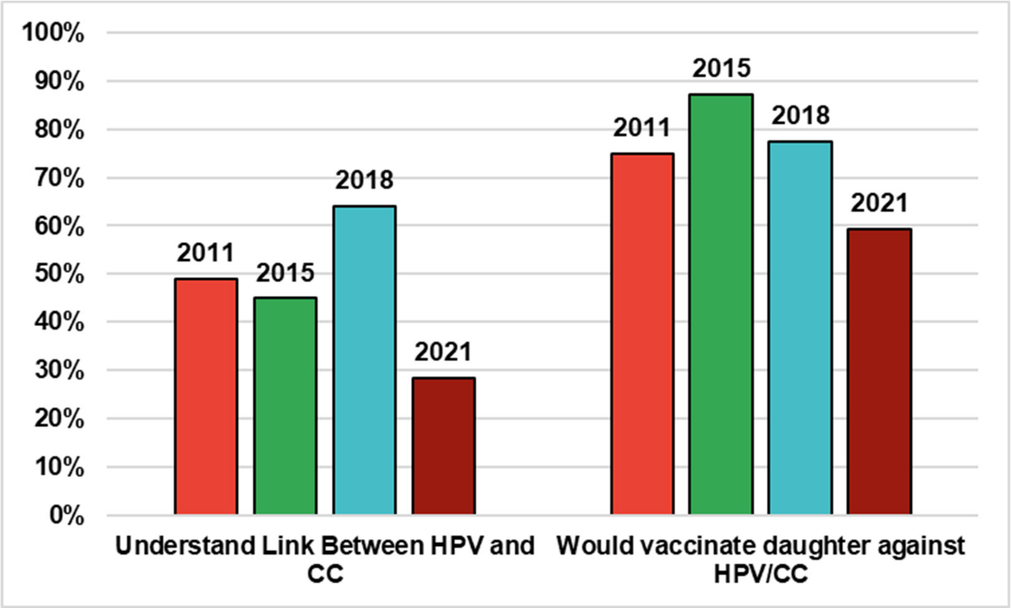

Supplement: Supplementary file 1 [file vaccines-13-00535-s001.zip › Supplemental Materials File S1.pdf]
