# Supplementary material for: Vaccine Confidence During Public Health Challenges and Prior to HPV Vaccine Introduction in Mali
Source: Vaccines (Basel). 2025 May 17;13(5):535. doi: 10.3390/vaccines13050535 (PMC12115454; doi:10.3390/vaccines13050535)
Supplement: Supplementary file 1 [file vaccines-13-00535-s001.zip › Supplemental Materials File S2 - Surveys.pdf]

Survey to be given to patients enrolled in the Corona Kele study

This survey can be offered to all adult patients presenting to the CSCOM, in French or in Bambara.
The CSCOM staff who give the questionnaire to patients must have received training on GAIA VF's vaccination information campaign, the objective of the CORONA KELE program, how to give this questionnaire and how to keep the documentation.

During the questionnaire, the notes for the interviewers are indicated on a grey background.
<u>They should NOT be read to patients.</u>

Before we begin, please verify that participants have been informed of the purpose of the survey and have signed the informed consent form. Indicate the date and patient ID below. The center codes are shown below.

This questionnaire is in six parts:

1. General information p1
2. Issues relating to vaccination in general ……................ p2
3. EPI questions p3
4. Questions relating to HPV and HPV vaccine p3-4
5. Questions relating to COVID-19 .... p4-5
6. Questions related to new COVID-19 vaccines p6

Date: |__|__|- |__|__| -|__|__|__|__|
DD MM YYYY

Identifier: |__|__| - |__|__|__| -|__|__|__|
Study Center Identifier

1. GENERAL INFORMATION

Health Center:
☐ CSREF (CSR)
☐ ASACOMSI (SIK)
☐ ASACOBA (BAN)
☐ ASACODJE (DJE)
☐ ASACOBOUL1 (BO1)
☐ ASACOBOUL2 (BO2)
☐ ASACODOU (DOU)
☐ ASACODJAN (ADI)
☐ ASACONORD (NOR)
☐ ASACOFADI (ADF)
☐ ASACOKOSA (KOS)
☐ ASACOS (SOD)
☐ ASACOSISOU (SIS)
☐ ASACOKENIERO (KEN)

Gender:
☐ Male
☐ Female

Age: ______________

How often do you attend a religious service?
☐ Weekly
☐ Monthly
☐ Only for religious holidays
☐ Never

Do you have children:
☐ Yes
☐ No

Level of education completed
☐ Elementary School
☐ High School
☐ College
☐ Graduate School
☐ None

2. QUESTIONS FOR VACCINES IN GENERAL

For each line, check the box that best reflects your feelings about vaccines in general:

	Completely Disagree	Disagree	No opinion	Agree	Completely Agree
1. I think vaccines are safe					
2. I think vaccines are important for children					
3. I think vaccines are effective					

4. Have you tried to get information about vaccines in the last 30 days? ☐Yes ☐ No
   If yes: Which vaccines?
   ☐ Vaccines in general
   ☐ HPV vaccines
   ☐ EPI vaccines
   ☐ COVID-19 vaccines
   ☐ Other (specify opposite)

   Allow the patient respond and check all the answers that apply.

5. Would you like to know more about vaccines? ☐ Yes ☐ No
   If yes: Which vaccines?
   ☐ Vaccines in general
   ☐ HPV vaccines
   ☐ EPI vaccines
   ☐ COVID-19 vaccines
   ☐ Other (specify opposite)

   Let the patient answer and check all the answers that apply.
   If the patient answers yes, please give information about the GAIA campaign, other information campaigns, or advise to contact their doctor.

6. Indicate how often you **follow the recommendations** of the people/entities below regarding vaccination.

	Always	Often	Sometimes	Never
The healthcare team of my CSCOM (doctors, nurses, midwives)				
Traditional healers				
Government and health authorities				
Local representatives (village chief, mayor, councilors)				
Religious leaders				
Celebrities (actors, singers, sportsmen)				
The head of the family				
My father				
Ma mother				
My friends/neighbors				
Other(specify):				

7. Indicate if you have been exposed to the following, and if they have influenced your confidence in vaccines.

Must have the pattern of the story-telling cloth and images of the posters used in the campaign. Check one answer per line.

	I have been exposed and my confidence in			Not exposed
	Lessened	Unchanged	Increased	
Discussion with CHWs on vaccination				
The story-telling cloth				
Radio announcement				
A poster				
Discussion with my CSCOM health staff				
Other (specify):				

Identifier: |__|__| - |__|__|__|__| - |__|__|__|

3. QUESTIONS RELATED TO THE EXPANDED PROGRAM ON IMMUNIZATION – for participants with children

8. Have your children been vaccinated under the expanded program on immunization?
If not, what are the reasons?
(Check all the reasons that apply)

☐Yes, all ☐Yes, some ☐ No ☐ I have no children

☐ My children are not at risk
☐ My children are not exposed
☐ I prefer my children to have the disease rather than the vaccine
☐ I'm worried about side effects / it's not safe
☐ I am against the vaccine in general
☐ Vaccines are not effective
☐ It's against my religion
☐ It takes too much time and money
☐ Other (specify opposite)

9. Do you know anyone who has been severely affected or has died from one of the diseases covered by routine EPI vaccinations?
Summarize the diseases and check yes if the patient identifies one or more disease(s)
Diphtheria, tetanus, pertussis, hepatitis B, yellow fever, measles, Haemophilus influenza type B (Hib), poliomyelitis, rotavirus, tuberculosis, meningitis, pneumococcal disease.

☐Yes ☐ No

10.	Have you had to cancel vaccination appointments because of the COVID-19 pandemic?	☐Yes ☐ No ☐ I don't know
11.	Have you had to cancel vaccination appointments for reasons other than the COVID-19 pandemic in 2020 and 2021?	☐Yes ☐ No ☐ I don't know
12.	Were you able to replace these cancelled appointments?	☐Yes ☐ No ☐ I don't know

4. HPV ISSUES

13.	Do you know about cervical cancer?	☐Yes ☐ No
14.	Have you ever heard of the HPV vaccine?	☐Yes ☐ No
15.	Do you think there is a link between the HPV vaccine and cervical cancer prevention?	☐Yes ☐ No ☐ I don't know
16.	Have you ever heard of the cervical cancer vaccine?	☐Yes ☐ No
17.	Would you agree to vaccinate your children against HPV / cervical cancer?	☐Yes ☐ No ☐ I don't know
18.	This vaccine protects against a sexually transmitted disease that causes cervical cancer. It is used in many countries and will soon be introduced to Mali. Does this information change your answer to the previous question?	☐Yes ☐ No ☐ I already knew

Identifier: |__|__| - |__|__|__|__| - |__|__|__|

19. Let's say you are the director of the CNI. Who should be given the vaccine to?

To babies	☐Yes	☐ No	☐ I don't know
To boys before marriage	☐Yes	☐ No	☐ I don't know
To girls before marriage	☐Yes	☐ No	☐ I don't know
To adult men	☐Yes	☐ No	☐ I don't know
To adult women	☐Yes	☐ No	☐ I don't know

5. COVID-19 ISSUES

20.	Do you know about COVID-19?	☐Yes ☐ No
21.	Do you know the cause of Covid-19?<br>***(The response must include infection with a virus)***	☐Yes ☐ No
22.	Do you think this is a major risk to the health of the community?	☐Yes ☐ No ☐ I don't know
23.	Do you think COVID-19 still exists in Mali?<br>If not, what are the reasons?<br>**(Check *all the reasons that apply)***	☐Yes ☐ No ☐ I don't know<br>☐ I never believed it<br>☐ We do not hear more about it<br>☐ Not enough cases<br>☐ Other (specify opposite)
24.	Do you think the COVID-19 virus survives in hot countries?	☐Yes ☐ No ☐ I don't know
25.	Do you think that the official figures relating to COVID-19 (number of sick and dead) reflect reality?	☐Yes ☐ No ☐ I don't know
26.	Do you fear a new wave of COVID-19 contamination?	☐Yes ☐ No ☐ I don't know
27.	Do you think COVID-19 can be a serious illness?	☐Yes ☐ No ☐ I don't know
28.	Who do you think COVID-19 affects **more often:**<br>Kids<br>The elderly<br>People who are overweight or obese<br>The poor<br>Rich people<br>The sickly	 ☐Yes ☐ No ☐ I don't know<br>☐Yes ☐ No ☐ I don't know<br>☐Yes ☐ No ☐ I don't know<br>☐Yes ☐ No ☐ I don't know<br>☐Yes ☐ No ☐ I don't know<br>☐Yes ☐ No ☐ I don't know
29.	Do you know someone who has been sick with the symptoms of COVID-19 but has not been tested?<br>***(Please explain the symptoms: difficulty breathing, flu-like illness, loss of smell or taste)***	☐ Yes ☐ No
30.	Do you know anyone who has been slightly ill with COVID-19?	☐ Yes ☐ No
31.	Do you know someone who has been seriously ill or has died as a result of Covid-19?<br>Yes, how many? Choose the nearest number	☐ Yes ☐ No<br>☐ 1 ☐5 ☐10 ☐20 or more
32.	Has your healthcare staff ever discussed COVID-19 prevention methods with you?	☐ Yes ☐ No ☐ I don't remember
33.	Currently, do you practice barrier gestures?	☐Yes ☐ No ☐ I don't know
34.	Do all the adults in your home have masks?	☐Yes ☐ No ☐ I don't know
35.	Have you ever been tested for COVID-19?	☐Yes ☐ No ☐ I don't know

Identifier: |__|__| - |__|__|__|__| - |__|__|__|

36. How do you greet your friends?

With an embrace	☐ Yes	☐ No
Shaking hands	☐ Yes	☐ No
By touching the point	☐ Yes	☐ No
By touching the elbow	☐ Yes	☐ No
Contactless	☐ Yes	☐ No

37. For each line, check the box that best reflects your habits **OVER THE PAST MONTH:**

	Not concerned	Never	Sometimes	Often	Always
I wash my hands when I get home					
I wear a mask outside the house					
I practice physical distancing					

38. Have you heard any rumors about COVID-19 and COVID-19 vaccines? If so, which ones and specify whether you believe in them or not.

(Let the patient answer and check the answers that apply)

	I've heard about ...			Never heard
	I believe in it	I don't believe it	It's possible	
The **virus** was created by a foreign government				
The **virus** was created by Bill Gates				
Vaccine contains microchips				
Vaccine contains magnets / makes my arm magnetic				
The vaccine will change my DNA				
The vaccine will give me COVID-19				
The vaccine will change me into a zombie				
All COVID-19 vaccines cause blood clots				
Once vaccinated, I am protected against COVID-19				
other:				

Identifier: |__|__| - |__|__|__|__| - |__|__|__|

6. ISSUES RELATED TO THE NEW VACCINES AGAINST COVID-19

39. Have you been vaccinated against COVID-19? ☐ Yes ☐ No
If yes: Date of first injection: _____ /20____
Month Year
Have you completed the vaccination? ☐ Yes ☐ No
(Please specify that vaccination Sinovac, SPUTNIK, Pfizer, Moderna or Astrazeneca is done in two doses, Johnson and Johnson in one dose)
Were you sick after the injection? If yes, specify symptoms: ☐ Yes ☐ No

---

40. Do you know anyone who has been vaccinated against COVID-19? ☐ Yes ☐ No
If so, was this person sick afterwards? ☐Yes ☐ No ☐ I don't know

---

41. If you have not been vaccinated, would you agree to be vaccinated against COVID-19? ☐Yes ☐ No ☐ I don't know

If you do not agree to be vaccinated, or if you are hesitant, what are the reasons?
(See all the reasons that apply and add the other reasons below)

☐ I don't think it will be prudent
☐ I'm not at risk enough to get covid-19
☐ I am worried about side effects
☐ I am against the vaccine in general
☐ I don't think it will work
☐ It's against my religion
☐ I'm sick / I don't feel well
☐ I want a different vaccine from the one offered to me
☐ Other (specify)

Would you agree to be vaccinated in exchange of money? ☐ Yes ☐ No ☐ I don't know

If you agree, or if you have already been vaccinated, what are the reasons?
(See all the reasons that apply and add the other reasons below)

☐ Protect myself from the disease
☐ Protect those around me (family / friends)
☐ Protecting seniors in my family
☐ Protecting children
☐ I follow the instructions
☐ Other (specify)

---

42. Who would you advise to receive the COVID-19 vaccine?

☐ To the elderly
☐ To my family
☐ To children
☐ To my friends / colleagues / neighbors
☐ I do not recommend it
☐ Other (specify)

End of questionnaire: Please give the patient a voucher for an upcoming visit to CSCOM.

Identifier: |__|__| - |__|__|__|__| - |__|__|__|

Sondage à donner aux patients recrutés dans l'étude Corona Kele

Ce sondage peut être proposé à tous les patients majeurs se présentant au CSCOM, en Français ou en Bambara.
Le personnel du CSCOM qui donne le questionnaire aux patients doit avoir reçu une formation sur la campagne d'information sur la vaccination de GAIA VF, l'objectif du programme CORONA KELE, comment asséner ce questionnaire et comment conserver la documentation.

Au cours du questionnaire, les notes destinées aux enquêteurs sont indiquées sur fond gris. Elles ne doivent pas être lues aux patients.

Avant de commencer, merci de vérifier que les participants ont été informés du but du sondage et ont signé le formulaire de consentement éclairé. Indiquer la date et l'identifiant patient ci-dessous. Les codes centres sont indiqués ci-dessous.

Ce questionnaire est en six parties :

1. Informations générales ……………………………………………………………… p1
2. Questions relatives à la vaccination en général ……………………………… p2
3. Questions relatives au PEV ………………………………………………………… p3
4. Questions relatives au HPV et au vaccin HPV …………………………………… p3
5. Questions relatives à la Covid-19 ………………………………………………… p4-5
6. Questions relatives aux nouveaux vaccins contre la Covid-19 ………… p6

Date : |__|__|- |__|__| -|__|__|__|__|
JJ MM AAAA

Identifiant : |__|__| - |__|__|__| - |__|__|__|
Etude Centre Identifiant

1. INFORMATIONS GENERALES

Centre de santé :
☐CSREF (CSR)
☐ASACOMSI (SIK)
☐ASACOBA (BAN)
☐ASACODJE (DJE)
☐ASACOBOUL1 (BO1)
☐ASACOBOUL2 (BO2)
☐ASACODOU (DOU)
☐ASACODJAN (DJA)
☐ASACONORD (NOR)
☐ASACOFADI (FAD)
☐ASACOKOSA (KOS)
☐ASACOS (SOD)
☐ASACOSISOU (SIS)
☐ASACOKENIERO (KEN)

Sexe :
☐Homme
☐Femme

Age : ______________

Avez-vous des enfants :
☐Oui
☐Non

Niveau d'éducation complété
☐Primaire
☐Collège
☐Lycée
☐Enseignement supérieur
☐Aucun

A quelle fréquence vous rendez-vous à un service religieux ?
☐Hebdomadaire
☐ Mensuel
☐Seulement pour les fêtes religieuses
☐Jamais

Identifiant : |__|__| - |__|__|__| - |__|__|__|

2. QUESTIONS RELATIVES AUX VACCINS EN GENERAL

Pour chaque ligne, cochez la case qui reflète au mieux votre sentiment par rapport aux vaccins en général :

	Pas du tout d'accord	Pas d'accord	Pas d'avis	D'accord	Très d'accord
1. Je pense que les vaccins sont sans danger					
2. Je pense que les vaccins sont importants pour les enfants					
3. Je pense que les vaccins sont efficaces					

4. Avez-vous essayé d'obtenir des informations sur les vaccins pendant les 30 derniers jours ?
Si oui : Quels vaccins ?

Laissez le patient répondre et cochez les réponses qui s'appliquent.

☐Oui ☐Non
☐Vaccins en général
☐Vaccins contre le HPV
☐Vaccins du PEV
☐Vaccins contre la Covid-19
☐Autres (précisez ci-contre)

5. Voudriez-vous en savoir plus sur les vaccins ?
Si oui : Quels vaccins ?

Laissez le patient répondre et cochez les réponses qui s'appliquent.
Si le patient répond oui, merci de donner les infos sur la campagne de GAIA ou d'autres campagnes d'information, ou conseillez de s'adresser à leur médecin.

☐Oui ☐Non
☐Vaccins en général
☐Vaccins contre le HPV
☐Vaccins du PEV
☐Vaccins contre la Covid-19
☐Autres (précisez ci-contre)

6. Indiquez la fréquence à laquelle vous **suivez les recommandations** des personnes / entités ci-dessous concernant la vaccination.

	Toujours	Souvent	Parfois	Jamais
L'équipe soignante de mon CSCOM (médecins, infirmières, sage femmes)				
Les guérisseurs traditionels				
Le gouvernement et les autorités de santé				
Les représentants locaux (chef du village, maire, conseillers)				
Les leaders religieux				
Les célébrités (acteurs, chanteurs, sportifs)				
Le chef de famille				
Mon père				
Ma mere				
Mes amis / voisins				
Autres (précisez):				

7. Indiquez si vous avez été exposé aux éléments suivants, et s'ils ont influencé votre confiance aux vaccins.

Montrez le motif du pagne et des images des posters utilisés dans la campagne. Cochez une réponse par ligne.

	J'ai été exposé et ma confiance à			Pas été exposé
	Diminué	Pas changé	Augmenté	
Discussion avec relais communautaire sur la vaccination				
Le pagne				
Annonce a la radio				
Un poster				
Discussion avec le personnel de sante de mon CSCOM				
Autre (précisez) :				

Identifiant : |__|__| - |__|__|__| - |__|__|__|

3. QUESTIONS RELATIVES AU PROGRAMME ELARGI DE VACCINATION – pour les participants avec enfants

8. Vos enfants ont-ils été vaccinés dans le cadre du programme élargi de vaccination ?
Si non, quelles sont les raisons ?
(Cochez toutes les raisons qui s'appliquent)

☐Oui, tous ☐Oui, pas tous ☐Non ☐Je n'ai pas d'enfant

☐Mes enfants ne sont pas à risque
☐Mes enfants ne sont pas exposés
☐Je préfère que mes enfants aient la maladie plutôt que le vaccin
☐Je suis inquiet des effets secondaires / ce n'est pas prudent
☐Je suis contre le vaccin en général
☐Les vaccins ne sont pas efficaces
☐C'est contre ma religion ☐Ça prend trop de temps et d'argent
☐Autre (précisez ci-contre)

9. Connaissez-vous quelqu'un qui a été atteint sévèrement ou est décédé d'un des maladies couvertes par les vaccinations de routine du PEV ?
Enumérez les maladies et cochez oui si le patient identifie une ou plusieurs maladie (s)
La diphtérie, le tétanos, la coqueluche, l'hépatite B, la fièvre jaune, la rougeole, l'Haemophilus influenza type B (Hib), la poliomyélite, le rotavirus, la tuberculose, la méningite, la maladie a pneumocoque.
☐Oui ☐Non

10. Avez-vous dû annuler des RDV de vaccinations à cause de la pandémie de Covid-19 ? ☐Oui ☐Non ☐Je ne sais pas

11. Avez-vous dû annuler des RDV de vaccinations pour d'autres raisons que la pandémie de Covid-19 en 2020 et 2021? ☐Oui ☐Non ☐Je ne sais pas

12. Avez-vous pu remplacer ces RDV annulés ? ☐Oui ☐Non ☐Je ne sais pas

4. QUESTIONS RELATIVES AU HPV

13. Connaissez-vous la maladie du cancer du col de l'utérus ? ☐Oui ☐Non

14. Avez-vous déjà entendu parler du vaccin contre le HPV ? ☐Oui ☐Non

15. Pensez-vous qu'il y ait un lien entre le vaccin contre le HPV et la prévention du cancer du col de l'utérus ? ☐Oui ☐Non ☐Je ne sais pas

16. Avez-vous déjà entendu parler du vaccin contre le cancer du col de l'utérus ? ☐Oui ☐Non

17. Seriez-vous d'accord pour vacciner vos enfants contre le HPV / cancer du col ? ☐Oui ☐non ☐Je ne sais pas

18. Ce vaccin protège contre une maladie sexuellement transmissible qui cause le cancer du col de l'utérus. Il est utilisé dans de nombreux pays, et va bientôt être introduit au Mali. Est-ce que cette information change votre réponse à la question précédente ? ☐Oui ☐Non ☐Je savais déjà

19. Imaginons que vous êtes le directeur du CNI. A qui doit-on donner le vaccin ?

Aux bébés	☐Oui	☐Non	☐Je ne sais pas
Aux garçons avant mariage	☐Oui	☐Non	☐Je ne sais pas
Aux filles avant mariage	☐Oui	☐Non	☐Je ne sais pas
Aux hommes adultes	☐Oui	☐Non	☐Je ne sais pas
Aux femmes adultes	☐Oui	☐Non	☐Je ne sais pas

5. QUESTIONS RELATIVES A LA COVID-19

20.	Connaissez- vous la Covid-19 ?	☐Oui ☐Non
21.	Connaissez-vous la cause de Covid-19 ?<br>***(La réponse doit inclure infection par un virus)***	☐Oui ☐Non
22.	Pensez-vous que c'est un risque majeur pour la santé de la communauté ?	☐Oui ☐Non ☐Je ne sais pas
23.	Pensez-vous que la Covid-19 existe toujours au Mali ?<br>Si non, quelles en sont les raisons ?<br>***(Cochez toutes les raisons qui s'appliquent)***	☐Oui ☐Non ☐Je ne sais pas<br>☐Je n'y ai jamais cru<br>☐On en entend plus parler<br>☐Pas assez de cas<br>☐Autre (précisez ci-contre)
24.	Pensez-vous que le virus de la Covid-19 survit dans les pays chauds ?	☐Oui ☐Non ☐Je ne sais pas
25.	Pensez-vous que les chiffres officiels relatifs à la Covid-19 (nombre de malades et de morts) reflètent la réalité ?	☐Oui ☐Non ☐Je ne sais pas
26.	Craignez-vous une nouvelle vague de contamination de Covid-19 ?	☐Oui ☐Non ☐Je ne sais pas
27.	Pensez-vous que la Covid-19 peut être une maladie grave ?	☐Oui ☐Non ☐Je ne sais pas
28.	Pensez-vous que la Covid-19 affecte **plus souvent** :	
	Les enfants	☐Oui ☐Non ☐Je ne sais pas
	Les personnes âgées	☐Oui ☐Non ☐Je ne sais pas
	Les personnes en surpoids ou obèse	☐Oui ☐Non ☐Je ne sais pas
	Les personnes pauvres	☐Oui ☐Non ☐Je ne sais pas
	Les personnes riches	☐Oui ☐Non ☐Je ne sais pas
	Les infirmes	☐Oui ☐Non ☐Je ne sais pas
29.	Connaissez-vous quelqu'un qui a été malade avec les symptômes de la Covid- 19 mais n'a pas été testé ?<br>***(Merci d'expliquer les symptômes : difficulté à respirer, syndrome grippal, perte de l'odorat ou du gout)***	☐Oui ☐Non
30.	Connaissez-vous quelqu'un qui a été légèrement malade de la Covid-19 ?	☐Oui ☐Non
31.	Connaissez-vous quelqu'un qui a été gravement malade ou est décédé des suites de la Covid-19 ?<br>Si oui, combien ? Choisissez le nombre le plus proche	☐Oui ☐Non<br>☐1 ☐5 ☐10 ☐20 ou plus
32.	Votre personnel de santé a-t-il déjà discuté avec vous des gestes barrières ?	☐Oui ☐Non<br>☐Je ne me rappelle pas
33.	Actuellement, pratiquez-vous les gestes barrières ?	☐Oui ☐Non ☐Je ne sais pas
34.	Les adultes de votre foyer ont-ils tous un masque ?	☐Oui ☐Non ☐Je ne sais pas
35.	Avez-vous déjà été testé(e) pour la Covid-19 ?	☐Oui ☐Non ☐Je ne sais pas
36.	Comment saluez-vous vos amis ?	
	Avec une accolade	☐Oui ☐Non
	En serrant la main	☐Oui ☐Non
	En touchant le point	☐Oui ☐Non
	En touchant le coude	☐Oui ☐Non
	Sans contact	☐Oui ☐Non

37. Pour chaque ligne, cochez la case qui reflète au mieux vos habitudes **AU COURS DU DERNIER MOIS** :

Identifiant : |__|__| - |__|__|__| - |__|__|__|

	Pas concerné	Jamais	Parfois	Souvent	Tout le temps
Je me lave les mains en arrivant à la maison					
Je porte un masque en dehors de la maison					
Je pratique la distanciation physique					

38. Avez-vous entendu des rumeurs à propos de la Covid-19 et des vaccins contre la Covid-19 ? Si oui, lesquelles et précisez si vous y croyez ou non.
    (Laissez le patient répondre et cocher les réponses qui s'appliquent)

	J'ai entendu parle de …			Jamais entendu
	J'y crois	Je n'y crois pas	C'est possible	
Le **virus** a été créé par un gouvernement étranger				
Le **virus** a été créé par Bill Gates				
Le vaccin contient des micropuces				
Le vaccin contient des aimants / rend mon bras magnétique				
Le vaccin va changer mon ADN				
Le vaccin va me donner la Covid-19				
Le vaccin va me changer en zombie				
Tous les vaccins contre la Covid-19 provoque des caillots sanguins				
Un fois vacciné(e), je suis protégé(e) contre la COVID-19				
Autre :				

[redacted]

39. Avez-vous été vacciné(e) contre la Covid-19 ? ☐Oui ☐Non
Si oui : Date de la première injection : _____ /20____
Mois Année
Avez-vous complété la vaccination ? ☐Oui ☐Non
(Merci de préciser que la vaccination Sinovac, SPUTNIK, Pfizer, Moderna ou Astrazeneca se fait en deux doses, Johnson and Johnson en une dose)
Avez-vous été malade après l'injection ? Si oui précisez : ☐Oui ☐Non

---

40. Connaissez-vous quelqu'un qui a été vacciné contre la Covid-19 ? ☐Oui ☐Non
Si oui, cette personne a-t-elle été malade après ? ☐Oui ☐Non ☐Je ne sais pas

---

41. Si vous n'avez pas été vacciné (e), seriez-vous d'accord pour vous faire vacciner contre la Covid-19 ? ☐Oui ☐Non ☐Je ne sais pas

Si vous n'êtes pas d'accord pour vous faire vacciner, ou si vous êtes hésitant(e), quelles en sont les raisons ?
(Cochez toutes les raisons qui s'appliquent et ajoutez ci-dessous les autres raisons)

☐Je ne pense pas que ce sera prudent
☐Je ne suis pas assez à risque de contracter la covid-19
☐Je suis inquiet des effets secondaires
☐Je suis contre le vaccin en général
☐Je ne pense pas que ça marchera
☐C'est contre ma religion
☐Je suis malade / je ne me sens pas bien
☐Je souhaite un vaccin différent de celui qui m'est proposé
☐Autres (précisez)

Accepteriez-vous de vous faire vacciner contre une somme d'argent ? ☐Oui ☐Non ☐Je ne sais pas

Si vous êtes d'accord, ou si vous avez déjà été vacciné(e), quelles en sont les raisons ?
(Cochez toutes les raisons qui s'appliquent et ajoutez ci-dessous les autres raisons)

☐Me protéger de la maladie
☐Protéger mon entourage (famille / amis)
☐Protéger les personnes âgées dans ma famille
☐Protéger les enfants
☐Je suis les consignes
☐Autres (précisez)

---

42. A qui conseilleriez-vous le vaccin contre la Covid-19 ?

☐Aux personnes âgées
☐A ma famille
☐Aux enfants
☐A mes amis / collègues / voisins
☐Je ne le conseille pas
☐Autre (précisez)

Fin du questionnaire : Merci de remettre au patient un bon pour une prochaine visite au CSCOM.

Identifiant : |__|__| - |__|__|__| - |__|__|__|

Welcome to GAIA VF's Training Workshops!

Thank you for participating in the vaccination training workshops. The workshops are intended to inform you about the risks and benefits of vaccination, the nature of the new vaccines that will be introduced in Mali, as well as the vaccines that are in development.

Four main themes will be addressed:

- Vaccination in General
- The Expanded Program on Immunization (EPI)
- The HPV vaccine
- New vaccines against COVID-19

Before the workshop begins, **please fill out the following questionnaire in the most honest way possible. Your answers are anonymous.**

GAIA VF will use your answers to identify training needs, staff confidence in vaccines and the effectiveness of these workshops. A similar questionnaire will be given to you at the end of the final workshop.

This questionnaire is divided into five parts:

1. EPI questions …………………………………….... p1
2. Questions relating to HPV and HPV vaccine p2
3. Questions relating to COVID-19 .... p3
4. Questions about new COVID-19 vaccines p4-5
5. General information p5

Thank you for your participation!
The GAIA VF team

1. ISSUES RELATED TO THE EXPANDED IMMUNIZATION PROGRAM

For each line, check the box that best reflects your feelings about vaccines in general:

	Completely Disagree	Somewhat Disagree	No opinion	Somewhat Agree	Completely Agree
1. I think vaccines are safe					
2. I think vaccines are important for children					
3. I think vaccines are effective					
4. I trust the government for health care recommendations.					

5. Do you have children? ☐ Yes ☐ No

If yes: Have they been vaccinated under the expanded program on immunization (EPI)? ☐ Yes ☐ No

6. **If your children have not been vaccinated**, what are the reasons? (Check all the reasons that apply and add the other reasons below)
   - ☐ My children are not at risk
   - ☐ My children are not exposed
   - ☐ I prefer that they have the disease rather than the vaccine
   - ☐ I'm worried about side effects / it's not safe
   - ☐ I am against the vaccine in general
   - ☐ Vaccines are not effective
   - ☐ It's against my religion
   - ☐ It takes too much time and money
   - ☐ Other (specify opposite)

7. Do you know someone who has been affected or died from an EPI disease?
   Examples of diseases covered by the EPI: diphtheria, tetanus, pertussis, hepatitis B, yellow fever, measles, Haemophilus influenza type B (Hib), poliomyelitis, rotavirus. tuberculosis, meningitis, pneumococcal disease, mumps
   - ☐ Yes, a member of my family
   - ☐ Yes, a friend
   - ☐ Yes, knowledge
   - ☐ Yes, a patient
   - ☐ No

8. Have you had to cancel or postpone appointments or vaccination events because of the COVID-19 pandemic? ☐ Yes ☐ No ☐ I am not affected

9. For each line, check the disease(s) that are prevented by each vaccine:

	OPV	Var	Rota	VAA	Penta	PCV13	Td	BCG	MenAfriVac
Whooping cough									
Diphtheria									
Yellow fever									
Haemophilus Influenzae type B									
Hepatitis B									
Pneumococcal disease									
Meningite									
Poliomyelitis									
Rotavirus									
Measles									
Tetanus									
Tuberculosis									

2. HPV ISSUES

9.	Have you ever heard of the HPV vaccine?	☐ Yes ☐ No
10.	Do you think there is a link between the HPV vaccine and cervical cancer prevention?	☐ Yes ☐ No ☐ I don't know
11.	Have you ever heard of the cervical cancer vaccine?	☐ Yes ☐ No
12.	Who do you think should receive an HPV vaccine?<br>(Check one answer for each category)	
	Boys before marriage	☐ Yes ☐ No ☐ I don't know
	Girls before marriage	☐ Yes ☐ No ☐ I don't know
	Adult men	☐ Yes ☐ No ☐ I don't know
	Adult women	☐ Yes ☐ No ☐ I don't know
13.	What type of vaccine is the HPV vaccine?	☐ Toxoid<br>☐ Living attenuated<br>☐ Inactivated<br>☐ Recombinant, subunit<br>☐ MESSENGER DNA or RNA<br>☐ I don't know

3. COVID-19 ISSUES

14. Do you think COVID-19 is a major public health risk? ☐ Yes ☐ No ☐ I don't know

15. Do you think COVID-19 still exists in Mali? ☐ Yes ☐ No ☐ I don't know
If not, what are the reasons (check all the reasons that apply)
☐ I never believed it
☐ We hear more about it
☐ Not enough cases
☐ Other (specify opposite)

16. Do you think the COVID-19 virus survives in hot countries? ☐ Yes ☐ No ☐ I don't know

17. Do you think that the official figures relating to COVID-19 (number of sick and dead) reflect reality? ☐ Yes ☐ No ☐ I don't know

18. Do you think COVID-19 can be a serious illness? ☐ Yes ☐ No ☐ I don't know

19. Who do you think COVID-19 affects **more often:**
Kids ☐ Yes ☐ No ☐ I don't know
The elderly ☐ Yes ☐ No ☐ I don't know
People who are overweight or obese ☐ Yes ☐ No ☐ I don't know
The poor ☐ Yes ☐ No ☐ I don't know
Rich people ☐ Yes ☐ No ☐ I don't know
The infirm ☐ Yes ☐ No ☐ I don't know

20. Do you know someone who has been ill or died from COVID-19? (check all that apply)
☐ Yes, a member of my family
☐ Yes, a friend
☐ Yes, an acquaintance
☐ Yes, a patient
☐ No

21. Do you think that some of your patients may have had COVID-19 but have not been tested? ☐ Yes ☐ No
If so, how much? Choose the nearest number. ☐ 1 ☐ 5 ☐10 ☐ 20 or more

22. Check the personal protective equipment that is available in your health center.
☐Gowns ☐ Masks
☐Gloves ☐ Face Shield
☐ Hydroalcoholic gel
☐ Other (specify opposite)

23. Have you ever been tested for COVID-19? ☐ Yes ☐ No ☐ I don't know

24. Do you think you have been exposed to Covid-19? ☐ Yes ☐ No ☐ I don't know

25. For each line, select the check box that best reflects your habits **OVER THE PAST MONTH:**

	All the time	Often	Sometimes	Occasionally	Never
I wash my hands at work					
I wash my hands when I get home					
I wear a mask in public places					
I wear a mask at work					
I talk to my patients about barrier gestures					

4. COVID-19 VACCINE ISSUES

26.	Have you been vaccinated against COVID-19?<br>If yes: Date of first injection: _____ /20____<br>Month Year	☐ Yes ☐ No
	Have you completed the vaccination?<br>Note that Sinovac, SPUTNIK, Pfizer, Moderna or Astrazeneca vaccination is completed in two doses, Johnson and Johnson in one dose.	☐ Yes ☐ No
	Were you sick after the injection? If yes, specify:	☐ Yes ☐ No
27.	Do you know anyone who has been vaccinated against Covid-19?<br>If so, did this person have any symptoms?	☐ Yes ☐ No<br>☐ Yes ☐ No ☐ I don't know
28.	If you have not been vaccinated, would you agree to be vaccinated against Covid-19?	☐ Yes ☐ No ☐ I don't know
	If you do **not agree** to be vaccinated, or if you are hesitant, what are the reasons?<br>*(Check all the reasons that apply and add the other reasons below*)	☐ I am not at risk of contracting Covid-19<br>☐ I don't think it will be prudent<br>☐ I am worried about side effects<br>☐ I am against the vaccine in general<br>☐ I don't think it will work<br>☐ COVID-19 vaccines are not effective<br>☐ It is against my religion<br>☐ It takes too much time and money<br>☐ Other (specify opposite)
	Would you agree to be vaccinated in exchange of money?	☐ Yes ☐ No ☐ I don't know
	If you **agree,** or if you have already been vaccinated, what are the reasons?<br>*(Check all the reasons that apply and add the other reasons below)*	☐ Protect myself from the disease<br>☐ Protect those around me (family / friends)<br>☐ Protecting seniors in my family<br>☐ Protecting children<br>☐ I follow the instructions<br>☐ Other (specify)
29.	To whom would you recommend the COVID-19 vaccine?	☐ To the elderly<br>☐ To my loved ones<br>☐ To my colleagues<br>☐ To my patients<br>☐ I do not recommend it
30.	Would you agree to participate in educational events to promote vaccines against COVID-19?	☐ Yes ☐ No<br>☐ Yes, if I had more information

31.	Who influences your decision to get vaccinated? (Check all the answers that apply and add the other reasons below)	☐ The health authorities (CNI, DRS, Ministry of Health)<br>☐ My supervisor<br>☐ My local political representative (chief, mayor, councilors)<br>☐ Family/friends<br>☐ A celebrity (singer, actor, sportsman)<br>☐ Traditional healer<br>☐ Professor / Scientist<br>☐ Other (specify opposite)
32.	Which COVID-19 vaccines are available in Mali?	☐ There is no vaccine against Covid-19 in Mali<br>☐ Moderna ☐ Astra Zeneca<br>☐ SinoVac ☐ Pfizer<br>☐ Sputnik ☐ Johnson and Johnson<br>☐ I don't know<br>☐ Other (specify):

5. GENERAL INFORMATION

Affiliation:
- ☐ CSREF
- ☐ ASACOMSI
- ☐ ASACOBA
- ☐ ASACODJE
- ☐ ASACOBOUL1
- ☐ ASACOBOUL2
- ☐ ASACODOU
- ☐ ASACODJAN
- ☐ ASACONORD
- ☐ ASACOFADI
- ☐ ASACOKOSA
- ☐ ASACOS
- ☐ ASACOSISOU
- ☐ ASACOKENIERO

Gender:
- ☐ Homme
- ☐ Femme

Age: ______________

Position:
- ☐ Doctor
- ☐ Nurse
- ☐ Pharmacist
- ☐ Midwife
- ☐ Matron
- ☐ Health Care Assistant
- ☐ Administrator
- ☐ Laboratory Technician
- ☐ Other:

Specialty:
- ☐ General medicine
- ☐ Pediatrics
- ☐ gynecology
- ☐ Surgery
- ☐ Ophthalmology
- ☐ Neurology
- ☐ ENT
- ☐ Infectious diseases
- ☐ Other:

____

Thank you for participating in GAIA VF's training workshops!

To enable us to improve these workshops in the future, and to understand the health staff's confidence in vaccines, please complete the following questionnaire. **Your answers are anonymous.**

This questionnaire is divided into five parts:

1. EPI questions …………………………………….... p1
2. Questions relating to HPV and HPV vaccine p2
3. Questions relating to COVID-19 .... p3
4. Questions about new COVID-19 vaccines p4-5
5. General information p5

Thank you for your participation!
The GAIA VF team

Please answer these questions first:

1.	Do you think the training workshops have changed your **confidence** in vaccines?	☐ An increase in trust<br>☐ No change<br>☐ A decrease in trust
2.	Do you think that the training workshops have increased your **knowledge** of vaccines?	☐ An increase in knowledge<br>☐ No change<br>☐ A decrease in knowledge
3.	Check the workshops you participated in.	☐ Vaccination<br>☐ EIP<br>☐ HPV<br>☐ COVID-19<br>☐ None

Bienvenue aux ateliers de formation de GAIA VF !

Merci de participer aux ateliers de formation sur la vaccination. Les ateliers ont lieu dans votre centre de santé et sont destinés à vous informer sur les risques et bénéfices de la vaccination, la nature des nouveaux vaccins qui vont être introduits au Mali, ainsi que les vaccins qui sont en développement.

Quatre grands thèmes seront abordés :

- La vaccination
- Le Programme Elargi de Vaccination (PEV)
- Le vaccin contre le papillomavirus, HPV
- Les nouveaux vaccins contre la Covid-19

Avant de commencer, **merci de bien vouloir remplir le questionnaire suivant**, de la façon la plus honnête possible. **Vos réponses sont anonymes.**

GAIA VF utilisera vos réponses pour identifier les besoins de formation, la confiance du personnel de santé envers les vaccins ainsi que l'efficacité de ces ateliers. Un questionnaire similaire vous sera remis à l'issue du quatrième atelier.

Ce questionnaire est en cinq parties :

1. Questions relatives au PEV ………………………………………………………………. p1
2. Questions relatives au HPV et au vaccin HPV ……………………………………. p2
3. Questions relatives à la Covid-19 ……………………………………………………. p3
4. Questions relatives aux nouveaux vaccins contre la COVID-19 …………. p4-5
5. Informations générales ………………………………………………………………….. p5

Merci de votre participation !
L'équipe de GAIA VF

[redacted]

Pour chaque ligne, cochez la case qui reflète au mieux votre sentiment par rapport aux vaccins en général :

	Pas du tout d'accord	Pas d'accord	Pas d'avis	D'accord	Très d'accord
1. Je pense que les vaccins sont sans danger					
2. Je pense que les vaccins sont importants pour les enfants					
3. Je pense que les vaccins sont efficaces					
4. Je fais confiance au gouvernement pour les recommandations de soins de santé.					

5. Avez-vous des enfants ? ☐Oui ☐Non
Si oui : Ont-ils été vaccinés dans le cadre du programme élargi de vaccination ? ☐Oui ☐Non

---

6. **Si vos enfants n'ont pas été vaccinés**, quelles en sont les raisons ? (Cochez toutes les raisons qui s'appliquent et ajoutez ci-dessous les autres raisons)

☐Mes enfants ne sont pas à risque
☐Mes enfants ne sont pas exposés
☐Je préfère que qu'ils aient la maladie plutôt que le vaccin
☐Je suis inquiet des effets secondaires / ce n'est pas prudent
☐Je suis contre le vaccin en général
☐Les vaccins ne sont pas efficaces
☐C'est contre ma religion
☐Ça prend trop de temps et d'argent
☐Autre (précisez ci contre)

---

7. Connaissez-vous quelqu'un qui a été affecté ou est décédé d'une maladie couverte par le PEV ?
Exemples de maladies couverte par le PEV: la diphtérie, le tétanos, la coqueluche, l'hépatite B, la fièvre jaune, la rougeole, l'Haemophilus influenza type B (Hib), la poliomyélite, le rotavirus. la tuberculose, la méningite, la maladie a pneumocoque., les oreillons

☐Oui, un membre de ma famille
☐Oui, un ami
☐Oui, une connaissance
☐Oui, un patient
☐Non

---

8. Avez-vous dû annuler ou reporter des RDV ou des événements de vaccinations à cause de la pandémie de Covid-19 ?

☐Oui ☐Non
☐Je ne suis pas concerné(e)

---

9. Pour chaque ligne, cochez la ou les maladie(s) qui sont prévenues par chaque vaccin :

	VPO	VAR	Rota	VAA	Penta	PCV13	TD	BCG	MenAfriVac
Coqueluche									
Diphtérie									
Fièvre Jaune									
Haemophilus Influenzae type B									
Hépatite B									
Maladie à pneumocoque									
Meningite									
Poliomyélite									
Rotavirus									
Rougeole									
Tétanos									

Tuberculose									

2. QUESTIONS RELATIVES AU HPV

9.	Avez-vous déjà entendu parler du vaccin contre le HPV ?	☐Oui ☐Non
10.	Pensez-vous qu'il y ait un lien entre le vaccin contre le HPV et la prévention du cancer du col de l'utérus ?	☐Oui ☐Non<br>☐Je ne sais pas
11.	Avez-vous déjà entendu parler du vaccin contre le cancer du col de l'utérus ?	☐Oui ☐Non
12.	Selon vous, qui devrait recevoir un vaccin contre le HPV ? (Cochez une réponse pour chaque catégorie)<br>Les garçons avant mariage<br>Les filles avant mariage<br>Les hommes adultes<br>Les femmes adultes	 ☐Oui ☐Non ☐Je ne sais pas<br>☐Oui ☐Non ☐Je ne sais pas<br>☐Oui ☐Non ☐Je ne sais pas<br>☐Oui ☐Non ☐Je ne sais pas
13.	Cochez la bonne réponse : Le vaccin contre le HPV est de type :	☐Toxoide<br>☐Vivant atténué<br>☐Inactivé<br>☐Recombinant, sous-unité<br>☐ADN ou ARN messager<br>☐Je ne sais pas

[redacted]

14. Pensez-vous que la Covid-19 est un risque majeur de santé publique ? ☐Oui ☐Non ☐Je ne sais pas

15. Pensez-vous que la Covid-19 existe toujours au Mali ? ☐Oui ☐Non ☐Je ne sais pas
Si non, quelles en sont les raisons (cochez toutes les raisons qui s'appliquent)
☐Je n'y ai jamais cru
☐On en entend plus parler
☐Pas assez de cas
☐Autre (précisez ci-contre)

16. Pensez-vous que le virus de la Covid-19 survit dans les pays chauds ? ☐Oui ☐Non ☐Je ne sais pas

17. Pensez-vous que les chiffres officiels relatifs à la Covid-19 (nombre de malades et de morts) reflètent la réalité ? ☐Oui ☐Non ☐Je ne sais pas

18. Pensez-vous que la Covid-19 peut être une maladie grave ? ☐Oui ☐Non ☐Je ne sais pas

19. Pensez-vous que la Covid-19 affecte **plus souvent** :
Les enfants ☐Oui ☐Non ☐Je ne sais pas
Les personnes âgées ☐Oui ☐Non ☐Je ne sais pas
Les personnes en surpoids ou obèse ☐Oui ☐Non ☐Je ne sais pas
Les personnes pauvres ☐Oui ☐Non ☐Je ne sais pas
Les personnes riches ☐Oui ☐Non ☐Je ne sais pas
Les infirmes ☐Oui ☐Non ☐Je ne sais pas

20. Connaissez-vous quelqu'un qui a été malade ou est décédé des suites de la Covid-19 ? (cochez toutes les réponses qui s'appliquent)
☐Oui, un membre de ma famille
☐Oui, un ami
☐Oui, une connaissance
☐Oui, un patient
☐Non

21. Pensez-vous que certains de vos patients ont pu avoir la Covid-19 mais n'ont pas été testés ? ☐Oui ☐Non
SI oui, combien ? Choisissez le nombre le plus proche. ☐1 ☐5 ☐10 ☐20 ou plus

22. Cochez les équipements de protection personnelle qui sont disponibles dans votre centre de santé.
☐Blouse ☐Masque
☐Gants ☐Visière
☐Gel hydroalcoolique
☐Autre (précisez ci-contre)

23. Avez-vous déjà été testé(e) pour la Covid-19 ? ☐Oui ☐Non ☐Je ne sais pas

24. Pensez-vous que vous avez été expose à la Covid-19 ? ☐Oui ☐Non ☐Je ne sais pas

25. Pour chaque ligne, cochez la case qui reflète au mieux vos habitudes **AU COURS DU DERNIER MOIS** :

	tout le temps	souvent	parfois	à l'occasion	jamais
Je me lave les mains au travail					
Je me lave les mains en arrivant à la maison					
Je porte un masque dans les lieux publics					
Je porte un masque au travail					
Je parle à mes patients des gestes barrières					

4. QUESTIONS RELATIVES AU VACCINS CONTRE LA COVID-19

26.	Avez-vous été vacciné(e) contre la Covid-19 ?<br>Si oui : Date de la première injection : _____ /20____<br>Mois Année<br>Avez-vous complété la vaccination ?<br>Notez que la vaccination Sinovac, SPUTNIK, Pfizer, Moderna ou Astrazeneca se complète en deux doses, Johnson and Johnson en une dose.<br><br>Avez-vous été malade après l'injection ? Si oui précisez :	☐Oui ☐Non<br><br>☐Oui ☐Non<br><br>☐Oui ☐Non
27.	Connaissez-vous quelqu'un qui a été vacciné contre la Covid-19 ?<br>Si oui, cette personne a-t-elle eu des symptômes ?	☐Oui ☐Non<br>☐Oui ☐Non ☐Je ne sais pas
28.	Si vous n'avez pas été vacciné (e), seriez-vous d'accord pour vous faire vacciner contre la Covid-19 ?<br><br>Si vous n'êtes **pas d'accord** pour vous faire vacciner, ou si vous êtes hésitant(e), quelles en sont les raisons ?<br>(cochez toutes les raisons qui s'appliquent et ajoutez ci-dessous les autres raisons)<br><br>Accepteriez-vous de vous faire vacciner contre une somme d'argent ?<br><br>Si vous êtes **d'accord**, ou si vous avez déjà été vacciné(e), quelles en sont les raisons ?<br>*(cochez toutes les raisons qui s'appliquent et ajoutez ci-dessous les autres raisons)*	☐Oui ☐Non ☐Je ne sais pas<br><br>☐Je ne suis pas à risque de contracter la Covid-19<br>☐Je ne pense pas que ce sera prudent<br>☐Je suis inquiet des effets secondaires<br>☐Je suis contre le vaccin en général<br>☐Je ne pense pas que ça marchera<br>☐Les vaccins Covid-19 ne sont pas efficaces<br>☐C'est contre ma religion<br>☐Ça prend trop de temps et d'argent<br>☐Autre (précisez ci contre)<br><br>☐Oui ☐Non ☐Je ne sais pas<br><br>☐Me protéger de la maladie<br>☐Protéger mon entourage (famille / amis)<br>☐Protéger les personnes âgées dans ma famille<br>☐Protéger les enfants<br>☐Je suis les consignes<br>☐Autres (précisez)
29.	A qui recommanderiez-vous le vaccin contre la Covid-19 ?	☐Aux personnes âgées<br>☐A mes proches<br>☐A mes collègues<br>☐A mes patients<br>☐Je ne le recommande pas
30.	Seriez-vous d'accord pour participer à des évènements d'éducation pour promouvoir les vaccins contre la Covid-19 ?	☐Oui ☐Non<br>☐Oui, si j'avais plus d'information
31.	Qui influence votre décision de vous faire vacciner ?<br>(cochez toutes les réponses qui s'appliquent et ajoutez ci-dessous les autres raisons)	☐ Les autorités de santé (CNI, DRS, Ministère de la sante)<br>☐Mon supérieur hiérarchique<br>☐ Mon représentant politique local (chef, maire, conseillers)<br>☐Famille / amis<br>☐Une célébrité (chanteur, acteur, sportif)

	☐Guérisseur traditionnel<br>☐Professeur / Scientifique<br>☐Autres (précisez ci-contre)
32. Quels vaccins contre la Covid-19 sont disponibles au Mali ?	☐Il n'y a pas de vaccin contre la Covid-19 au Mali<br>☐Moderna ☐Astra Zeneca<br>☐SinoVac ☐Pfizer<br>☐Spoutnik ☐Johnson and Johnson<br>☐Je ne sais pas<br>☐Autre (précisez) :

5. INFORMATIONS GENERALES

Affiliation :
☐CSREF
☐ASACOMSI
☐ASACOBA
☐ASACODJE
☐ASACOBOUL1
☐ASACOBOUL2
☐ASACODOU
☐ASACODJAN
☐ASACONORD
☐ASACOFADI
☐ASACOKOSA
☐ASACOS
☐ASACOSISOU
☐ASACOKENIERO

Sexe :
☐Homme
☐Femme

Age : ______________

Poste :
☐Docteur
☐Infirmier(ère)
☐Pharmacien(ne)
☐Sage-femme
☐Matrone
☐Aide-soignante
☐Administrateur
☐Technicien de laboratoire
☐Autre :

Spécialité :
☐Médecine générale
☐Pédiatrie
☐Gynécologie
☐Chirurgie
☐Ophtalmologie
☐Neurologie
☐ORL
☐Maladies infectieuses
☐Autre :
____

Merci d'avoir participé aux ateliers de formation de GAIA VF !

Pour nous permettre d'améliorer ces ateliers dans le futur, et de comprendre la confiance du personnel de santé envers les vaccins, merci de bien vouloir remplir le questionnaire qui suit. **Vos réponses sont anonymes.**

Ce questionnaire est en cinq parties :

1. Questions relatives au PEV …………………………………………………………….. p1
2. Questions relatives au HPV et au vaccin HPV ………………………………….. p2
3. Questions relatives à la Covid-19 ……………………………………………………. p3
4. Questions relatives aux nouveaux vaccins contre la COVID-19 …………. p4-5
5. Informations générales ……………………………………………………………………. p5

Merci de votre participation !
L'équipe de GAIA VF

Merci de bien vouloir répondre d'abord à ces questions :

1.	Pensez-vous que les ateliers de formation ont changé votre **confiance** envers les vaccins ?	☐Augmentation de la confiance<br>☐Pas de changement<br>☐Diminution de la confiance
2.	Pensez-vous que les ateliers de formation ont augmenté votre **connaissance** des vaccins ?	☐Augmentation de la connaissance<br>☐Pas de changement<br>☐Diminution de la connaissance
3.	Cochez les ateliers auxquels vous avez participé.	☐Vaccination<br>☐PEV<br>☐HPV<br>☐Covid-19<br>☐Aucun
