# Supplementary material for: Vaccine Confidence During Public Health Challenges and Prior to HPV Vaccine Introduction in Mali
Source: Vaccines (Basel). 2025 May 17;13(5):535. doi: 10.3390/vaccines13050535 (PMC12115454; doi:10.3390/vaccines13050535)
Supplement: Supplementary file 1 [file vaccines-13-00535-s001.zip › Supplemental Materials File S3 - IRB Approvals.pdf]

Ethical & Independent Review Services
A Shared Commitment to Ethical Research

# IRB APPROVAL CERTIFICATION

## Initial Review - Study

E&I IRB #2 - IRB00007807
Roster dated January 12, 2021

### IRB Signature

Signature Judith Cohen
Judith Cohen, PhD, IRB Vice-Chair

By TM
Teresa Majors

9/3/2021
Date

### Review and Approval Information

E&I Study Number 21116 - 01
Review Process Expedited 7

Approval Date Thursday, September 2, 2021
Expiration Date Thursday, September 1, 2022 at 11:59 PM

**This document certifies the IRB's approval and acknowledgment, per 45 CFR 46 (2018 Requirement), of the bolded items identified under "Documents Approved" and "Document Acknowledged" to be conducted by the named Principal Investigator.**

**A waiver of consent process was granted under 45 CFR 46.116(d) for illiterate persons.**

**A waiver of the requirement for documentation of informed consent is granted according to 45 CFR 46.117(c)(2) for Key Opinion Leaders.**

**All translated documents must be submitted to the IRB, including certifications, prior to enrollment of Non-English speaking subjects.**

**As the study is to also take place outside the United States, this approval for the conduct of study procedures is conditioned upon adherence to all local laws and regulations, including additional review and approval by local ethics committees, and/or adherence to privacy and security laws, (e.g., GDPR), as applicable to each location where conduct will occur.**

**In accordance with §46.109(f), the requirement for continuing review does not apply to this study. IRB approval will not expire on the stated expiration date, however a continuing review check-in process must be completed on or before the stated expiration date. Any changes to research activity continue to require IRB approval prior to implementation, except when necessary to eliminate apparent immediate hazards to the subject.**

**NOTE: Subjects must be asked for their consent using the most recently approved, stamped version(s). All IRB approved consent documents are version controlled and may not be modified in any way without prior IRB approval. Use of an unapproved document may constitute non-compliance.**

### Study

Corona Kele: Assessment of vaccine confidence among healthcare workers, key opinion leaders and within the peri-urban and rural communities in Bamako

Client GAIA Vaccine Foundation
Sponsor Merck and Co

### Grant Number and/or Title

60675; Corona Abana!: Evaluation of vaccine confidence in the peri-urban community of Bamako Mali, among patients, medical providers and key opinion leaders, in anticipation of Cervical Cancer (HPV) & COVID-19 vaccination campaigns

### Principal Investigator

Anne S. De Groot, MD

E&I PI Number 7972 - 001

### Address

GAIA Vaccine Foundation
188 Valley Street, Suite 424
Providence, RI 02909

### Performance Sites

GAIA Vaccine Foundation, 188 Valley Street, Suite 424, Providence, RI 02909

Centre de Sante de Reference Commune 1, Bamako, Mali, West Africa

Centre de Sante de Keniero Commune de Keniero - Si, Cercle De Kati - Region de Koulikoron Siby, Mali, West Africa

This is a multi-sided document.

Ethical & Independent Review Services
*A Shared Commitment to Ethical Research*

# IRB APPROVAL CERTIFICATION

## Initial Review - Study

E&I IRB #2 - IRB00007807
Roster dated January 12, 2021

| Documents Approved | Document # | Version | Date |
|---|---|---|---|
| Protocol | | 1.2 | 07/22/21 |
| Consent form for Patients (E&I 09/03/2021) - English | Annex I | 1 | 8/27/2021 |
| Consent form for Healthcare Workers (E&I 09/03/2021) - English | Annex II | 1 | 8/27/2021 |
| Recruitment Plan | | | eFile 7.26.21 |
| COVID Poster, "Together let's respect these measures to avoid..." | | eFile 1 | eFile 8.12.21 |
| COVID Poster, "How is the Coronavirus transmitted?" | | eFile 2 | eFile 8.12.21 |
| COVID Poster, "Ensemble respectons ces mesures pour éviter..." | | eFile 1 | eFile 7.26.21 |
| COVID Poster, "Comment se Transmet le Coronavirus?" | | eFile 2 | eFile 7.26.21 |
| Patient Questionnaire - English | Annex III | | eFile 7-22-21 |
| Questionnaire for Healthcare Personnel - English | Annex IV | | eFile 7-22-21 |
| Patient Questionnaire - French | Annexe III | | eFile 7-22-21 |
| Questionnaire for Healthcare Personnel - French | Annexe IV | | eFile 7-22-21 |

| Documents Acknowledged | Document # | Version | Date |
|---|---|---|---|
| Monthly Report Form for Study Sites - English | Annex VI | | eFile 7-22-21 |
| Monthly Report Form for Study Sites - French | Annexe VI | | eFile 7-22-21 |

## Stipulations of Approval

1. No subjects may be involved in any study procedure prior to the IRB approval date or after the expiration date, unless otherwise stated in this letter. Investigators and sponsors are responsible for initiating Continuing Review proceedings.
2. All protocol modifications must be IRB approved prior to implementation. This includes any addition or change of recruitment materials, change of investigator, or performance site address. (Exception: If necessary to eliminate apparent immediate hazard to subjects.)
3. Report to E&I within five working days of learning if any of the following occur:
   - Unanticipated problems involving risk to human subjects or others;
   - Unanticipated Serious Adverse Events and Safety Reports;
   - Protocol deviations, violations, and exceptions that impact subject welfare or safety or study integrity including changes intended to reduce immediate risk to subjects;
   - Use of an investigational product in an emergency situation; and
   - Claims for compensation or for medical care for research-related injury.
4. Advertising and recruitment materials must be approved by E&I prior to use or publication.

## Copies to:

Sarah Beseme, sarah.beseme@gaiavaccine.org

END

This is a multi-sided document.

Ethical & Independent Review Services
*A Shared Commitment to Ethical Research*

August 23, 2022

Anne S. De Groot, MD
CEO/CSO
GAIA Vaccine Foundation
188 Valley Street, Suite 424
Providence, RI 02909

RE: Corona Kele: Assessment of vaccine confidence among healthcare workers, key opinion leaders and within the peri-urban and rural communities in Bamako
E&I ID# 21116

Dear Anne De Groot:

Thank you for submitting the requested check-in report to communicate your progress on this study. In accordance with 45 CFR 46.109(f), continuing review by the IRB is not required. An administrative review of your reported progress has been performed to assist in assuring adherence to your reporting obligations. No further action is required at this time. You may proceed with the study. Please keep this document with your study records.

| | |
|---|---|
| E&I Assigned Study ID: | 21116 - 02 |
| Date of Initial Approval: | September 2, 2021 |
| Date of Check-In: | September 1, 2023 |

**Please continue to**:

1. Report any protocol violations, and or serious and related unanticipated problems involving risks to your subjects or others in a timely manner.
2. Submit any desired modifications for review and consideration prior to carrying out such changes.
3. Submit a new check-in report of your study activities before the check-in date noted above.

We appreciate having the opportunity to work with you on this project. Please reach out to our administration if you need any assistance during your study.

Sincerely,

Leslie Wilson

Leslie Wilson
Organizational Official
Ethical & Independent Review Services

**UNIVERSITE DES SCIENCES,**
**DES TECHNIQUES ET DES TECHNOLOGIES DE BAMAKO**

FACULTE DE MEDECINE ET D'ODONTO-STOMATOLOGIE
FACULTE DE PHARMACIE/ BP 1805, *BAMAKO – MALI*

☎ : (223) 20 22 52 77 ☎ : (223) 20 22 96 58

**N°2021/ 182 /CE/USTTB**

Bamako, le 27 juillet 2021

*Le Président du Comité d'Ethique de l'USTTB*

*A*

**Professeur Ousmane KOITA**

Cher Professeur,

J'ai le plaisir de vous informer que le comité d'Ethique de l'USTTB approuve définitivement votre protocole de recherche intitulé «**Evaluation de la confiance aux vaccins parmi les agents de santé, leaders d'opinion et au sein des communautés péri-urbaines et rurales de Bamako**» ayant constaté l'effectivité de la prise en compte des différentes recommandations faites.

Cette approbation est valable du **27 juillet 2021 au 26 juillet 2022**. Elle sera renouvelée après le dépôt du rapport annuel.

Le Comité d'Ethique de l'USTTB vous souhaite plein succès dans vos recherches.

P/LE PRESIDENT P.O
LE VICE- PRESIDENT

Comité d'Ethique de l'Université des Sciences des Techniques et des Technologies de Bamako * Le Président

Prof. Amadou DIALLO
